# The role and clinical potential of RNA modifications in bladder cancer

**DOI:** 10.14440/bladder.2024.0062

**Published:** 2025-03-06

**Authors:** Yuqing Wu, Fenghao Zhang, Yufan Ying, Jiangfeng Li, Xiangyi Zheng, Liping Xie

**Affiliations:** 1 Department of Urology, The First Affiliated Hospital, Zhejiang University School of Medicine, Hangzhou, Zhejiang 310003, China; 2 Ningbo Institute of Urology, Ningbo, Zhejiang 315000, China

**Keywords:** RNA modifications, m6A methylation, Bladder cancer, Drug resistance, Prognosis

## Abstract

**Background:**

Bladder cancer (BC) represents a common malignancy and is characterized by high heterogeneity and complex biological behaviors, which pose substantial challenges to its effective treatment. Mounting evidence highlights the pivotal roles of ribonucleic acid (RNA) modifications, particularly N6-methyladenosine, alongside others such as 1-methyladenosine, 5-methylcytosine, N4-acetylcytidine, and 7-methylguanosine, in the regulation of the proliferation, migration, drug resistance, and immune evasion of BC cells.

**Objective:**

This article comprehensively reviewed the regulatory mechanisms and biological impacts of these RNA modifications in BC, with a focus on the interplay between RNA modifications and immune evasion, as well as their emerging roles in precision medicine. By looking into these underexplored areas, this work provided novel insights into RNA modifications as diagnostic markers, prognostic indicators, and therapeutic targets, paving the way for advancements in BC precision medicine.

**Conclusion:**

RNA modifications impact key processes such as proliferation, migration, drug resistance, and immune evasion of BC cells. Targeting RNA modification pathways offers promising strategies for enhancing the efficacy of current treatments for and overcoming drug resistance of BC.

## 1. Introduction

Bladder cancer (BC) represents one of the most common malignancies affecting the urinary system across the globe, with persistently high incidence and recurrence rates.^1^ Despite advances in diagnostic and therapeutic strategies in recent years, the high heterogeneity, complex biological behaviors, and variable patient responses to conventional chemotherapy and immunotherapy continue to present significant challenges to its effective treatment.^2^,^3^ Understanding the related mechanisms, as well as identifying novel biomarkers and pathways involved, is crucial for improving survival in BC patients.

In cancer molecular biology, RNA modifications have been increasingly recognized as critical regulators in the development and progression of malignancies. These modifications involve chemical alterations to RNA molecules that affect their structure or stability, thereby influencing a wide array of biological processes, including gene expression, signal transduction, cell proliferation, and anti-apoptotic responses. Among these modifications, N6-methyladenosine (m6A) has been most extensively studied and is seen as a key player in several types of cancer. In BC, m6A modification has been shown to significantly influence the expression of oncogenes and tumor suppressors, and enhance tumor cell adaptability to hostile microenvironments.^4^

Apart from m6A, other RNA modifications, such as 1-methyladenosine (m1A), 5-methylcytosine (m5C), and pseudouridine, also contribute meaningfully to the biological features of BC cells.^5^ For instance, m1A-modified messenger RNA (mRNA) and long non-coding RNA (lncRNA) have been found to be biomarkers for predicting overall survival in BC patients,^6^ whereas m5C modification promotes cancer cell survival by stabilizing mRNA.^7^ The distribution, specificity, and dynamic regulation of these diverse RNA modifications across different stages of BC are different dimensions of the multilayered mechanisms underlying BC progression.

Ribonucleic acid (RNA) modifications serve various functions in the initiation and progression of BC, offering potential approaches to biomarker identification, individualized treatment, and drug resistance mitigation/suppression.^8^ Clinically, RNA modification factors are of substantial value in the diagnosis, prognostic assessment, and targeted therapy of BC.^9^ Modulating these factors could improve the effectiveness of chemotherapy and immunotherapy.

While the significance of RNA modifications in cancer biology has been well acknowledged, their specific roles and the mechanisms in BC remain under-studied. In particular, how RNA modifications regulate the tumor immune microenvironment (TIM) – such as by facilitating immune evasion – warrants further investigation. Previous studies have suggested that certain microbiota, such as *Porphyromonas somerae*, contribute to BC development by inducing chronic inflammation or altering host immune responses.^10^ These mechanisms may involve immune evasion and microenvironmental regulation.

In the realm of precision medicine, RNA modifications have shown preliminary promise as biomarkers for cancer therapy. For instance, studies on testicular cancer have demonstrated that specific RNA biomarkers, such as microRNAs (miRNAs), can predict therapeutic response and disease relapse.^11^ However, these advances have yet to be fully translated into BC treatment. The heterogeneity of BC further underscores the potential of RNA modifications as targets for precision therapy, yet the current literature remains fragmented and a comprehensive review is lacking.

This review systematically looked at the biological mechanisms and clinical potential of RNA modifications in BC. Different from existing studies, this work emphasized the role of RNA modifications in the modulation of TIM and their applications in precision medicine, including the prediction of immunotherapeutic responses and management/control of drug resistance. By integrating the multifaceted regulatory effects and clinical implications of RNA modifications, we aimed to provide a broader perspective and in-depth insights to advance both their basic research and translational application in BC.

## 2. Types and regulatory mechanisms of RNA modifications in BC

### 2.1. Enzymes and regulatory mechanisms of m6A methylation in BC

#### 2.1.1. m6A methylation-related enzymes

m6A is one of the most abundant post-transcriptional RNA modifications identified to date and plays a vital role in the regulation of gene expression, RNA stability, and cellular functions. Notably, the overall level of m6A is elevated in BC.^12^ The regulation of m6A is controlled by a series of key enzymes, including methyltransferases (“writers”), demethylases (“erasers”), and binding proteins (“readers”). Together, these enzymes form a complex regulatory network that influences the post-transcriptional fate of RNA.

2.1.1.1. Writers

Methyltransferase-like protein (METTL) 3 and METTL14 are core components of the m6A methyltransferase complex. They work together to catalyze the addition of methyl groups to specific sites on RNA. This modification typically occurs at the “DRACH” consensus sequence (D = G, A, or U; R = A or G; H = A, C, or U),^13^-^16^ which is predominantly located in the 5’ untranslated region (UTR), 3’ UTR, and exons of mRNAs.^17^ METTL3, a central component of the m6A methylation complex, participates in multiple RNA-related processes, including pre-mRNA splicing, 3’ end processing, nuclear export, translational regulation, mRNA decay, and miRNA processing.^18^ High METTL3 expression has been linked to poor prognosis in BC.^19^ Wilms’ tumor 1-associating protein (WTAP), as an auxiliary factor, enhances the stability and catalytic activity of the m6A methylation complex^20^ and reportedly was upregulated in BC tissues.^21^ In addition, RNA-binding protein 15 contains RNA-binding domains that allow it to recognize specific RNA sequences, thereby aiding in the targeting of the m6A methylation complex to designated RNA molecules. METTL16, another member of the METTL protein family, has also been reported to catalyze m6A modifications on mRNA.^17^

2.1.1.2. Erasers

Fat mass and obesity-associated protein (FTO) and human alkyl homolog H5 (ALKBH5) are the primary demethylases responsible for removing m6A modifications, and thereby regulating RNA stability and translational efficiency. FTO, an α-ketoglutarate-dependent dioxygenase, specifically removes m6A marks, primarily acting within the nucleus, and is often associated with nuclear speckles. The sequence motifs for m6A removal sites typically follow a “GAC” and “AAC” consensus pattern, and these sites are found in the 5’ UTR, 3’ UTR, exons, and termination codons.^22^ ALKBH5, another α-ketoglutarate-dependent dioxygenase, also performs demethylation functions and participates in the regulation of RNA metabolism and function.^23^

2.1.1.3. Readers

YTH m6A RNA binding protein (YTHDF) 1, YTHDF2, and YTHDF3 are key m6A-binding proteins that specifically recognize m6A-modified RNA through particular domains, affecting its stability and translation.^24^ The YTHDF protein family, characterized by their YT521 homology (YTH) domains,^16^,^21^,^25^ recognizes m6A-modified RNA and regulates various mRNAs linked to tumor progression, with significant biological implications. YTH domain-containing protein YTHDC1 and YTHDC2 are additional m6A “reader” proteins, possessing YTH domains similar to those of the YTHDF family.^15^ YTHDC1 is modulated under high glucose conditions and is downregulated in BC tissues, being correlated with cancer prognosis.^26^ YTHDC1 can regulate nuclear RNA splicing, selective polyadenylation, nuclear export, and decay.^27^ The insulin-like growth factor 2 mRNA-binding protein (IGF2BP) family, also serving as m6A readers, modulates mRNA stability and translation by recognizing m6A modifications.^28^ IGF2BP1 contains six classical RNA-binding domains, including two RNA recognition motifs and four hnRNP-K homology domains (KH domains), with KH3 and KH4 deemed crucial for interactions with target genes.^29^ In addition, fragile X messenger ribonucleoprotein 1 (FMR1) and HIV-1 Rev interacting protein, heterogeneous nuclear ribonucleoproteins A2/B1 (HNRNPA2B1) have been identified to be essential m6A readers, playing critical roles in regulating immune cell infiltration and serving as predictors for the efficacy of immunotherapy.^30^

#### 2.1.2. Regulatory mechanisms of m6A methylation in BC

During the m6A modification, methyltransferases, such as METTL3 and METTL14, bind to specific RNA sequences and catalyze the methylation, adding a methyl group to the N6 position of adenosine residues. This process is regulated by various cellular signals and environmental factors, achieving a dynamic equilibrium. For example, studies have shown that melittin selectively downregulates METTL3 expression in BC cells, thereby modulating m6A levels.^13^ On the other hand, hypoxic conditions can regulate m6A levels by suppressing METTL16 expression.^17^ Demethylation is carried out by FTO and ALKBH5, which recognize and remove m6A modifications on RNA, thereby modulating RNA stability and translational efficiency.^24^

The recognition of m6A modifications is premised on the functioning m6A-binding proteins. For instance, YTHDF1 enhances the translation efficiency of CUB domain-containing protein 1 mRNA by recognizing its m6A modification.^31^ In contrast, YTHDF2 binds to the coding sequence region of the target gene mRNA, promoting the degradation of these transcripts.^14^,^32^ YTHDF3 also recognizes m6A modifications, facilitating the translation of specific targets, such as integrin alpha-6.^33^ This recognition process not only influences RNA stability but also dictates the fate of RNA in terms of translation and degradation. Similarly, IGF2BP family proteins play a part in this pathway. For example, IGF2BP2 binds to m6A-modified RNA, specifically stabilizing neuropilin-1 mRNA.^34^ IGF2BP3 enhances the stability and expression of high-mobility group box 1 (HMGB1) by binding to HMGB1 mRNA.^35^

Other m6A readers also contribute significantly to RNA regulation. FMR1 is primarily involved in the nuclear export of m6A-modified mRNA and interacts with YTHDF1 and YTHDF2 to maintain the stability of mRNA targets.^30^ HNRNPA2B1, meanwhile, promotes mRNA splicing and nucleocytoplasmic transport, and enhances the processing of primary miRNAs, thus playing a key role in RNA metabolism. In addition, HNRNPA2B1 has been implicated in promoting lymph node metastasis and recurrence in certain cancers.^30^

An m6A scoring system, developed through research, has revealed prognostic differences among BC patients. Patients with higher m6A scores tend to have better prognoses, whereas their low m6A score counterparts have poorer outcomes. The m6A score is also associated with immune cell infiltration. A high m6A score typically correlates with greater infiltration of CD4 and CD8 T cells and with better clinical responses to immunotherapy with anti-cytotoxic T-lymphocyte-associated protein 4 (CTLA-4).^33^

### 2.2. Other RNA modifications involved in BC

#### 2.2.1. m5C modification of RNA

m5C refers to/is the resultant product of the addition of a methyl group to the C atom at the fifth position of cytidine nucleoside. In 1950, Wyatt GR first confirmed the presence of m5C in nucleic acids.^44^ Since then, m5C, as an RNA modification, has been extensively found in various RNA species, including mRNA, transfer RNA (tRNA), ribosomal RNA (rRNA), and non-coding RNA. This modification is closely associated with RNA stability and translation efficiency.^45^ Similar to m6A modification, m5C modification is also regulated by different writers, erasers, and readers.

*2.2.1.1. Writers*

The “writers” of m5C can be divided into two categories: the NOP2/Sun RNA methyltransferase (NSUN) family and the deoxyribonucleic acid (DNA) methyltransferase (DNMT) family. The NSUN family includes NSUN1 (p120, known as NOP2 in yeast), NSUN2, NSUN3, NSUN4, NSUN5 (known as Rcm1 in yeast), NSUN6, and NSUN7. Meanwhile, the DNMT family includes DNMT1, DNMT3A, DNMT3B, and TRDMT1 (also known as DNMT2).^46^-^48^ NSUN2 is the earliest reported and studied member of the NSUN family. Initially, tRNA-specific methyltransferase 4 (Trm4) was found to be involved in the regulation of tRNA methylation modifications in yeast. Subsequently, NSUN2 was identified as the homologous protein of Trm4 in animals.^49^,^50^ NSUN2 is responsible for m5C modification on mRNA, particularly near the translation initiation site, the 3’ UTR, and regions near argonaute-binding sites. In BC, NSUN2 targets m5C sites in the 3’ UTR of hepatoma-derived growth factor (HDGF) mRNA, thereby stabilizing HDGF mRNA.^7^ DNMT2 is another m5C writer that has been primarily studied apart from NSUN2. It specifically methylates cytosine in the anticodon loop of tRNA.^51^ In addition, DNMT2 can catalyze the methylation of mRNA,^52^ although its role in BC has not been studied to date.

*2.2.1.2. Erasers*

The ten-eleven translocation (TET) family proteins (TET1, TET2, and TET3) function as the main “erasers” by demethylating m5C sites. They convert RNA 5-methylcytidine to 5-hydroxymethylcytidine, 5-formylcytidine, and 5-carboxycytidine through oxidation. AlkB homolog (ALKBH) 1/Arabidopsis homolog (ABH1) is another known eraser. The m5C modification can be further oxidized by α-ketoglutarate and iron (II)-dependent dioxygenase ALKBH1/ABH1, generating 5-formyl cytosine at this position.^53^ Compared to m5C readers, reports on the role and mechanisms of m5C erasers in BC are relatively scarce.

*2.2.1.3. Readers*

Aly/REF export factor (ALYREF) and Y-box binding protein (YBX) 1 are recognized as the main “reader” proteins for m5C,^54^,^55^ with other reader proteins including radiation sensitive 52, methyl-CpG-binding domain protein 1 (MBD) 1, MBD2, MBD3, MBD4, methyl CpG binding protein 2, Nei-like DNA glycosylase 1, endonuclease III-like protein 1, single-strand-selective monofunctional uracil-DNA glycosylase 1, ubiquitin-like, containing PHD and RING finger domains (UHRF) 1, UHRF2, uracil-DNA glycosylase, zinc finger and BTB domain-containing (ZBTB) 33, ZBTB38, and ZBTB4.^46^-^48^

YBX 1 is an m5C-binding protein that contains a cold shock domain (CSD) capable of sequence-dependent recognition of the RNA CA(U/C)C motif. The tryptophan 65 residue within the CSD forms a CH-π interaction with m5C, allowing YBX1 to recruit ELAV-like RNA binding protein 1 and maintain the stability of its target mRNA, thereby affecting RNA metabolism.^54^

ALYREF, also known as THOC4, is another specific m5C-binding protein that recognizes and binds to m5C regions in RNA through the lysine 171 residue, thereby regulating mRNA nuclear export.^56^ Wang *et al*.^57^ demonstrated that ALYREF specifically binds to highly-methylated m5C sites within the mRNAs of RABL6 and TK1, promoting the splicing and stability of these transcripts, which are associated with the malignancy of BC. In addition, Wang *et al*.^58^ found that ALYREF2 binds m5C in the 3’ UTR to stabilize pyruvate kinase M2 (PKM2) mRNA in BC.

#### 2.2.2. m1A modification of RNA

The occurrence of m1A in RNA was first reported by Dunn DB in 1961.^59^ Later, Dominissini *et al*.^60^ and Li *et al*.^61^ confirmed the presence of a dynamic m1A methylome in eukaryotic mRNA.

m1A is a crucial post-transcriptional modification that is predominantly found within the structure of tRNA.^62^ The tRNA methyltransferase 6 non-catalytic subunit (TRMT6)/tRNA methyltransferase 61A (TRMT61A) complex mediates m1A modification at the 58^th^ nucleotide position (m1A58) of cytoplasmic tRNA, with TRMT61A serving as the catalytic subunit and TRMT6 facilitating tRNA binding. m1A58 is the main form of m1A modification in eukaryotic tRNA. ALKBH1 and ALKBH3, members of the AlkB protein family, catalyze the oxidative demethylation of m1A in tRNA. In addition, Yin *et al*.^63^ discovered that m1A modification also occurs on mRNA and lncRNA, primarily within the “CAGGC” motif.

#### 2.2.3. N4-acetylcytidine modification of RNA

N4-acetylcytidine (ac4C) is a type of RNA modification initially identified in bacterial tRNA anticodon loops^64^ and subsequently found in eukaryotic serine and leucine tRNAs, as well as 18S rRNA.^65^ In 2018, Arango *et al*.^66^ confirmed that ac4C is also present as an epitranscriptomic modification on mRNA.

At present, N-acetyltransferase 10 (NAT10) is the first and only known enzyme to catalyze ac4C modifications in eukaryotic RNA. NAT10, a member of the Gcn5-related N-acetyltransferase (GNAT) family, consists of 1,025 amino acids and includes conserved domains for N-acetyltransferase activity, adenosine triphosphate (ATP)/guanosine triphosphate binding, and ATPase activity.^67^ NAT10 catalyzes ac4C formation by binding to the D-arm regions of tRNA serine and tRNA leucine, with the THUMPD1 protein’s THUMP domain facilitating this modification. NAT10 also catalyzes ac4C modification at position 1,842 in the terminal helix of 18S rRNA, participating in rRNA processing and ribosome assembly.^68^ In eukaryotic cells, NAT10 adds ac4C modifications to specific mRNA sites, enhancing mRNA stability and translation efficiency.^66^ For example, NAT10 promotes cyclin-dependent kinase inhibitor p21 mRNA stability and translation efficiency by adding ac4C to its 3’ UTR, whereas chloride intracellular channel 3 (CLIC3) inhibits NAT10, reducing ac4C modification and, thus, p21 mRNA stability.^69^ NAT10-mediated ac4C modifications primarily occur near wobble positions in coding mRNAs, where they enhance decoding efficiency. Xie *et al*.^70^ also observed ac4C modification in the vicinity of termination codons in cytosine-rich sequences, particularly “CXX” motifs. NAT10 directly interacts with these CXX motifs through its GNAT and helicase domains, catalyzing ac4C modification and recruiting heterogeneous nuclear ribonucleoprotein Q and thyroid hormone receptor-associated protein 3 to enhance mRNA stability and protect it from nuclease degradation. Wang *et al*.^71^ demonstrated that NAT10 participates in the ac4C modification of B-cell CLL/lymphoma 9-like (BCL9L), SRY-related HMG-box 4 (SOX4), and serine/threonine-protein kinase (AKT1) mRNAs in BC cells, enhancing their stability and translation efficiency through wobble position acetylation.

#### 2.2.4. 7-methylguanosine modification of RNA

METTL 1, also known as tRNA (guanine-N7)-methyltransferase, catalyzes 7-methylguanosine (m7G) modification on human tRNA, particularly at position 46 within the tRNA variable loop, by forming a complex with WD repeat domain 4.^72^ Ying *et al*.^73^ found that METTL1-mediated m7G tRNA modification regulates the translation of epidermal growth factor receptor (EGFR) and EGF-containing fibulin-like extracellular matrix protein 1 (EFEMP1), promoting BC progression through the METTL1-m7G-EGFR/EFEMP1 axis. METTL1-mediated m7G tRNA modification also regulates tRNA abundance and selectively enhances the translation of transcripts with codons frequently decoded by m7G-modified tRNA, suggesting that METTL1’s activity is dynamically regulated by cellular or environmental factors.^73^,^74^ Moreover, Xie *et al*.^75^ discovered that METTL1 can regulate miRNA by methylating primary miR-760 with m7G, thereby promoting its processing.

#### 2.2.5. Other RNA modifications

Begley *et al*.^76^ identified a methylation modification in tRNA mediated by a human TRM9-like protein, a human tRNA methyltransferase containing an S-adenosylmethionine-dependent methyltransferase domain. This modification affects protein translation by regulating the cell cycle through interactions with the tumor suppressor Lin-9, a component of the DREAM MuvB core complex.

## 3. Role of RNA modifications in the biology of BC cells

### 3.1. m6A modifications and the biology of BC cells

#### 3.1.1. Impact of m6A modifications on proliferation and migration of BC cells

m6A modifications play a multifaceted role in promoting the proliferation and migration of BC cells, regulating these processes through various pathways (Table 1). Studies have demonstrated that the m6A methyltransferase METTL3 is upregulated in BC, facilitating tumor cell proliferation and invasion.^18^ METTL3 regulates oncogenes such as MYC, ALF transcription elongation factor 4, and v-rel avian reticuloendotheliosis viral oncogene homolog A by modulating their m6A levels. This modification enhances the expression of these genes, thereby activating the proliferation and survival capacity of BC cells.^18^

**Table 1:** Functions, mechanisms, and effects of key enzymes on tumor behaviors in BC

Enzyme	Functions and mechanisms	Effects on BC cell behaviors	References
METTL3	Activates PI3K/AKT/mTOR, MYC and NF-κB pathways via m6A modification; promotes maturation of miR-221/222 and miR-146a-5p; collaborates with YTHDF1 and YTHDF2 to degrade tumor suppressors (e.g., SETD7, KLF4, RRAS)	Increases cell proliferation, migration, and invasion; enhances chemotherapy resistance and immune evasion	13,18,19,24,36
METTL14	Stabilizes USP38 to inhibit EMT and migration; is linked with Notch1 signaling to suppress tumor-initiating cell self-renewal	Reduces BC cell migration and invasion; increases sensitivity to treatments	16,37
WTAP	Adds m6A modification to NRF2 mRNA, enhancing NRF2 stability; is linked to SYTL1 m6A modification, which supports resistance to NK cell anti-tumor activity	Promotes cell proliferation, migration, and anti-apoptotic ability; increases drug resistance	12,21
FTO	Demethylates and reduces stability of oncogenes such as MALAT1 and NOTCH1; downregulates MMP9 and CCND1 through miR-5581-3p; stabilizes STAT3	Effects on cancer behaviors can vary depending on specific cellular contexts; in some cases, FTO may act as a tumor suppressor, whereas in others, it may promote cancer progression	22,38,39
ALKBH5	Demethylates ITGA6 mRNA, reducing its stability and protein expression	Decreases cell proliferation and migration; may enhance sensitivity to chemotherapy drugs	40
YTHDF1	Recognizes m6A modification on GRIN2D and NRF2, enhancing their stability; regulates aerobic glycolysis by binding m6A-marked GRIN2D	Increases cell proliferation, glycolysis, and migration	41
YTHDF2	Recognizes and degrades tumor suppressor mRNAs (e.g., RIG-I, SETD7, KLF4); suppresses immune response; forms m6A regulatory axis with YTHDC1 and METTL3	Promotes cell proliferation and survival, and increases chemotherapy resistance	14,42
YTHDC1	Stabilizes PTEN mRNA, inhibiting PI3K/AKT pathway; low expression reduces immune response and increases tumor invasiveness	Suppresses BC cell proliferation; enhances cisplatin sensitivity. At high glucose, YTHDC1 downregulation promotes glycolysis and tumor progression	43

Abbreviations: Akt: Protein kinase B; ALKBH5: Human alkyl homolog H5; BC: Bladder cancer; CCND1: Cyclin D1; EMT: Epithelial-mesenchymal transition; FTO: Fat mass and obesity-associated protein; GRIN2D: Glutamate [NMDA] receptor subunit epsilon-4; ITGA6: Integrin alpha-6; KLF4: Krüppel-like factor 4; MALAT1: Metastasis-associated lung adenocarcinoma transcript 1; METTL: Methyltransferase-like protein; miR: MicroRNA; MMP9: Matrix metalloproteinase-9; mRNA: Messenger RNA; mTOR: Mechanistic target of rapamycin; m6A: N6-methyladenosine; NF-κB: Nuclear factor kappa-light-chain-enhancer of activated B cells; NK: Natural killer; NOTCH1: Neurogenic locus notch homolog protein 1; NRF2: Nuclear factor erythroid 2-related factor 2; PI3K: Phosphatidylinositol 3-kinase; PTEN: Phosphatase and tensin homolog; RIG-I: Retinoic acid-inducible gene I; RRAS: Rat sarcoma virus-related protein; SETD7: SET domain containing lysine methyltransferase 7; STAT3: Signal transducer and activator of transcription 3; SYTL1: Synaptotagmin-like protein 1; USP38: Ubiquitin-specific peptidase 38; WTAP: Wilms’ tumor 1-associating protein; YTHDC1: YTH domain-containing protein 1; YTHDF: YTH N6-methyladenosine RNA binding protein.

Moreover, METTL3 also regulates the maturation of miR-146a-5p through m6A modification, further activating the NUMB endocytic adaptor protein (NUMB)/neurogenic locus notch homolog protein (NOTCH) 2 signaling pathway to promote cancer cell growth. miR-146a-5p’s suppression of NUMB activates the oncogenic NOTCH2 signaling pathway, revealing METTL3’s critical role as an oncogenic factor in BC and enhancing BC cells’ proliferative and invasive abilities.^13^ In addition, METTL3 and YTHDF2 facilitate the degradation of rat sarcoma virus-related (RRAS) mRNA through m6A modification, destabilizing RRAS. As a key regulator of anti-proliferative signaling, the loss of RRAS disrupts proliferation inhibition in BC cells, accelerating malignant progression.^42^

Conversely, METTL16 mediates m6A modification at the 3’ UTR region of prostate transmembrane protein, androgen-induced 1 (PMEPA1) mRNA, reducing PMEPA1 mRNA stability and decreasing its protein expression, thereby inhibiting cell proliferation.^17^ However, FTO exhibits a dual role in BC, acting as both an oncogenic and a tumor-suppressive factor. Some studies depicted that FTO promotes oncogenicity through demethylation that stabilizes transcripts such as signal transducer and activator of transcription 3, thereby enhancing cancer cell proliferation and migration.^39^ Conversely, other studies indicated that FTO possesses a tumor-suppressive potential, as it reduces the stability of metastasis-associated lung adenocarcinoma transcript 1 (MALAT1) and NOTCH1 by decreasing their m6A methylation, consequently limiting cancer cell proliferation and migration.^22^ This dual function of FTO in BC may depend on specific molecular targets and the cellular microenvironment, underscoring its complex regulatory role within the cancer context.

m6A modifications not only influence BC cell proliferation but also significantly enhance cancer cell migration and metastatic potential. The METTL3/YTHDF2-mediated m6A regulatory axis promotes BC cell invasiveness by degrading the tumor-suppressive factors SET domain containing lysine methyltransferase 7 and Krüppel-like factor 4, thereby diminishing their tumor-suppressive functions.^36^ In addition, YTHDF1 stabilizes glutamate [NMDA] receptor subunit epsilon-4 (GRIN2D) mRNA by binding to its m6A sites in BC, preventing GRIN2D degradation and elevating its expression. GRIN2D enhances glycolysis in cancer cells, promoting cell invasion and migration, which consequently contributes to the malignancy of tumor cells.^41^

Moreover, m6A modification stabilizes the lncRNA BLACAT3, which in turn recruits the YBX3 protein to the nucleus. YBX3 binds to the neutrophil cytosolic factor 2 promoter, activating the nuclear factor kappa-light-chain-enhancer of activated B cells (NF-κB) signaling pathway. This cascade of events not only enhances the invasiveness of BC cells but also promotes angiogenesis, creating a favorable tumor microenvironment (TME) that supports metastasis.^79^

Beyond regulating cancer cell proliferation and migration, m6A modifications also substantially impact BC cells’ responses to the immune system. WTAP enhances m6A modification on synaptotagmin-like protein 1 (SYTL1) mRNA, facilitating its recognition and degradation by YTHDF2, which in turn downregulates SYTL1 expression.^21^ Typically, the upregulation of SYTL1 boosts natural killer (NK) cell activity by increasing receptors such as NK group 2 member D protein, natural killer protein (NKp) 30, and NKp44, thereby enhancing NK cell activation. However, WTAP-mediated downregulation of SYTL1 enables BC cells to evade NK cell attacks, providing a proliferation and invasion advantage in the immune environment.^21^ In addition, YTHDC1 deficiency may modulate immune cell infiltration in the TME, particularly promoting the accumulation of M2 macrophages, which indirectly supports BC growth and invasion.^27^

Furthermore, YTHDF2 suppresses the immune response by binding to and promoting the degradation of DDX58 mRNA, which encodes retinoic acid-inducible gene I (RIG-I), a critical immune regulator. Reduced RIG-I expression diminishes apoptosis and enhances BC cell proliferation.^14^

#### 3.1.2. Regulation of signaling pathways by m6A modifications in BC

In BC, m6A modifications further promote cancer cell proliferation and migration by regulating key signaling pathways. The phosphatidylinositol 3-kinase (PI3K)/protein kinase B (AKT)/mechanistic target of the rapamycin (mTOR) pathway, a central axis for cell growth and survival, is significantly influenced by m6A-modifying factors. YTHDF1 and METTL3 activate the PI3K/AKT/mTOR pathway by upregulating the ribophorin II (RPN2) gene, thereby enhancing the proliferative capacity and anti-apoptotic properties of cancer cells.^24^ In contrast, YTHDC1 modulates this pathway by upregulating phosphatase and tensin homolog (PTEN) expression in an m6A-dependent manner, thereby inhibiting PI3K/AKT signaling.^43^

m6A modifications also affect BC cells’ invasiveness through the regulation of epithelial-mesenchymal transition (EMT)-related genes. As an m6A methyltransferase, METTL14 stabilizes mRNA expression of ubiquitin-specific peptidase 38 (USP38), thereby inhibiting BC cell migration and EMT. Specifically, METTL14 upregulates USP38, which enhances the expression of tumor suppressor genes that inhibit EMT-related transcription factors such as Snail, Slug, and zinc finger E-box-binding homeobox 1.^16^ This mechanism demonstrates how METTL14 hinders the expression of EMT markers (e.g., matrix metalloproteinase-9) in BC cells, creating a barrier to cancer cell migration and dissemination.

Moreover, YTHDC1 plays a significant role in regulating glycolytic pathways, further driving BC malignancy. Under high-glucose conditions, YTHDC1 expression decreases, leading to increased expression of glucose transporter 3 (GLUT3), which enhances the glycolytic capacity of BC cells. A GLUT3 accelerates cancer cell energy metabolism, promoting more active growth and invasion. YTHDC1 contributes to BC progression by regulating a feedback loop involving GLUT3 and RING finger 183, thereby enhancing glucose metabolism and advancing tumor development.^26^

In summary, m6A modifications promote BC cell proliferation, migration, and immune evasion through the regulation of multiple biological pathways, including the PI3K/AKT/mTOR pathway, EMT, and glycolysis. These complicated signaling mechanisms collectively enhance the malignancy of BC, laying the bedrock for further research into the role of m6A modifications in BC and uncovering the therapeutic potential of targeting m6A-related factors in the future.

### 3.2. Other RNA modifications and the biology of BC cells

#### 3.2.1. Other RNA modifications in BC cell proliferation and apoptosis

Chen *et al*.^7^ demonstrated that m5C modification is associated with the onset and progression of BC. The sustained expression of HDGF is dependent on the m5C catalytic activity of NSUN2 and the m5C-binding ability of YBX1, which jointly stabilize HDGF mRNA (Table 2).

**Table 2:** Functions and mechanisms of enzymes regulating other ribonucleic acid modifications in bladder cancer

Modification type	Category	Enzyme	Functional roles	Molecular mechanisms	Related genes	References
m5C	Writer	NSUN2	Drives BC pathogenesis	Targets m5C sites in the 3’ UTR of HDGF mRNA to stabilize HDGF mRNA	HDGF	7
	Reader	YBX1	Drives BC pathogenesis	Recognizes m5C-modified RNA through W65 in its CSD to form CH-π; directly recruits and interacts with ELAVL1 to enhance the stability of its m5C-modified mRNA	HDGF	7,77
		ALYREF	Drives BC pathogenesis	Recognizes and binds to m5C regions in RNA through the K171 residue, regulating mRNA nuclear export	PKM2, HIF-1α	56,58
			Drives BC pathogenesis	Binds to highly methylated m5C sites to promote mRNA splicing and transcript stability	RABL6, TK1	57

m1A	Writer	TRMT6/TRMT61A	Promotes cell proliferation and cellular stress resistance	Mediates m1A modification at the 58th nucleotide position (m1A58) of cytoplasmic tRNA	tRF	63,78
	Eraser	ALKBH3	Promotes cancer cell invasion	Catalyzes oxidative demethylation of m1A in tRNA	-	78

ac4C	Writer	NAT10	Promotes cell proliferation	Promotes p21 mRNA stability and translation efficiency by adding ac4C to its 3’ UTR; inhibited by CLIC3	p21, CLIC3	69
			Promotes cisplatin chemoresistance	Binds and stabilizes AHNAK mRNA by protecting it from exonucleases	AHNAK, HNRNPQ, THRAP3, NFKB1 (p65)	70
			Promotes cell proliferation, migration, invasion, survival, and stem-cell-like properties	Promotes mRNA stability and translation efficiency	BCL9L, SOX2, AKT1	71

m7G	Writer	METTL1/WDR4	Promotes tumorigenesis, progression, and invasion	Modulates the translation of EGFR/EFEMP1	EGFR, EFEMP1	74
		METTL1	Promotes cell proliferation and metastasis	Promotes the processing of miR-760 and contributes to the degradation of ATF3 mRNA	ATF3	75
			Promotes cell proliferation, migration, and invasion	Upregulates EGFR/EFEMP1 protein expression	EGFR, EFEMP1	73

Abbreviations: ac4C: N4-acetylcytidine; AHNAK: Neuroblast differentiation-associated protein; AKT1: Serine/threonine-protein kinase; ALKBH3: AlkB homolog 3, alpha-ketoglutarate-dependent dioxygenase; ALYREF: Aly/REF export factor; ATF3: Activating transcription factor 3; BC: Bladder cancer; BCL9L: B-cell CLL/lymphoma 9 like; CLIC3: Chloride intracellular channel 3; CSD: Cold shock domain; EFEMP1: EGF-containing fibulin-like extracellular matrix protein 1; EGFR: Epidermal growth factor receptor; ELAVL1: ELAV-like RNA binding protein 1; HDGF: Hepatoma-derived growth factor; HIF-1α: Hypoxia-inducible factor 1-alpha; HNRNPQ: Heterogeneous nuclear ribonucleoproteins Q; K171: Lysine 171 residue; METTL1: Methyltransferase-like 1; miR: MicroRNA; mRNA: Messenger RNA; m1A: 1-methyladenosine; m5C: 5-methylcytosine; NAT10: N-acetyltransferase 10; NFKB1 (p65): Nuclear factor kappa b subunit 1; NSUN2: NOP2/Sun RNA methyltransferase 2; PKM2: Pyruvate kinase M2; p21: Cyclin-dependent kinase inhibitor; RABL6: RAB, member RAS oncogene family-like 6; RNA: Ribonucleic acid; SOX2: SRY-related HMG-box; THRAP3: Thyroid Hormone Receptor Associated Protein 3; TK1: Thymidine kinase 1; TRMT6: tRNA methyltransferase 6 non-catalytic subunit; TRMT61A: tRNA Methyltransferase 61A; tRNA: Transfer RNA; tRF: tRNA-derived fragments; UTR: Untranslated region; WDR4: WD repeat domain 4; W65: Tryptophan 65 residue; YBX1: Y-box binding protein 1.

The m5C modification promotes tumor initiation and progression in BC by stabilizing HDGF mRNA, ensuring its high expression in BC tissues.^78^ ALYREF enhances BC cell proliferation through glycolysis mediated by PKM2.^56^ The m1A modification, regulated by TRMT6/TRMT61A, not only promotes BC cell proliferation but also increases resistance to cellular stress.^78^ High METTL1 expression in BC is associated with poor prognosis and silencing or depletion of METTL1 significantly suppresses the proliferation of BC cell lines, such as T24 and UM-UC3.^73^,^75^ What is more, CLIC3 inhibits NAT10 function by interacting with it, leading to lowered ac4C modification and stability of p21 mRNA. These events result in reduced p21 expression, which subsequently affects cell proliferation.^62^,^69^-^71^

#### 3.2.2. Effects of other RNA modifications on invasion, migration, and distant metastasis of BC cells

The depletion of TRMT6/TRMT61A slows the average migration speed of 5,637 cells, potentially impacting cell migration ability.^78^ BC cells with high m5C expression, particularly muscle-invasive BC cells, exhibit higher methylation levels and increased invasiveness.^57^ Ying *et al*.^73^ demonstrated that the loss of METTL1 significantly suppresses BC cell migration, invasion, and distant metastasis. Cytoplasmic FMR1-interacting protein 1 (CYFIP1), a tumor suppressor, is implicated in tumor migration and invasion by influencing actin nucleation and signaling pathways, such as the β1-integrin/focal adhesion kinase/non-receptor tyrosine kinase axis. Studies have shown that abnormal expression of CYFIP1 is linked to metastasis in breast cancer, suggesting that m7G modification may regulate distant metastasis by affecting genes such as CYFIP1. However, research on this in BC remains limited.^72^ Moreover, NAT10-mediated ac4C modification of mRNAs, such as BCL9L, SOX4, and AKT1, in BC cells enhances their stability and translation efficiency, promoting migration, invasion, and distant metastasis in BC cell lines.^71^

## 4. Impact of RNA modifications on drug sensitivity in BC cancer

### 4.1. m6A and drug sensitivity

#### 4.1.1. Regulation of cisplatin sensitivity by m6A modifications

Studies have shown that high expression levels of YTHDF1 and METTL3 significantly enhance cisplatin resistance in BC cells. YTHDF1 increases RPN2 expression through m6A modification, which subsequently activates the PI3K/AKT/mTOR signaling pathway, thereby endowing cells with greater survival capabilities and increasing cisplatin resistance.^24^ Under conditions of low YTHDC1 expression, BC cells exhibit even higher resistance to cisplatin. YTHDC1 inhibits PI3K/AKT pathway activity by regulating PTEN expression, which is crucial for cisplatin treatment. When YTHDC1 is downregulated, PTEN expression decreases, leading to enhanced AKT signaling, higher cell survival rates, and increased cisplatin resistance.^43^ Besides, METTL16 influences the sensitivity of BC cells to cisplatin by regulating autophagy-related pathways through m6A modification of PMEPA1 mRNA.^17^

#### 4.1.2. Regulation of the sensitivity to other chemotherapeutic agents by m6A modifications

High expression of YTHDF2 in BC is associated with increased resistance to chemotherapeutic drugs such as fluorouracil, gemcitabine, and mitomycin C. YTHDF2 degrades DDX58 mRNA, which encodes RIG-I, thereby inhibiting the RIG-I signaling pathway and reducing apoptosis sensitivity. This mechanism diminishes the efficacy of chemotherapeutic drugs, enhancing cancer cell survival and promoting chemoresistance.^14^ As an m6A demethylase, low expression of FTO reduces BC cell sensitivity to chemotherapy. Upregulating FTO to modulate m6A demethylation levels, particularly for oncogenes such as MALAT1 and NOTCH1, can help increase cancer cell sensitivity to chemotherapy.^80^ Furthermore, research indicated that METTL3 enhances BC cell resistance to melittin by regulating the expression of miR-146a-5p.^13^

These findings underscore the critical role of m6A modifications in the changed sensitivity of BC to chemotherapeutics, providing potential therapeutic targets for overcoming chemoresistance in BC treatment.

### 4.2. Effects of other RNA modifications on drug resistance and chemotherapy sensitivity in BC

Monshaugen *et al*.^78^ demonstrated that depletion of TRMT6/TRMT61A sensitizes 5,637 BC cells to tunicamycin, a cellular stress inducer, indicating TRMT6/TRMT61A role in stress response and tolerance. Xie *et al*.^70^ revealed that cisplatin induces NAT10-mediated ac4C modification in BC cells through the NF-κB pathway. Specifically, NF-κB p65 directly binds to the promoter region of NAT10 to activate its transcription, which in turn enhances DNA damage repair by protecting neuroblast differentiation-associated protein mRNA from exonuclease degradation, thereby increasing cisplatin resistance in BC cells. Existing studies have shown that the m5C reader protein YBX1 enhances cisplatin sensitivity through autophagy in non-small cell lung cancer.^77^ However, YBX1 has been reported to increase cisplatin resistance in BC.^81^

## 5. The role of RNA modifications in the clinical application potential of BC

### 5.1. Potential clinical applications of m6A modifications in BC

m6A modifications hold significant clinical promise for BC in the areas of diagnosis, prognostication, and treatment. Numerous m6A-modifying factors, through their regulation of cancer cell proliferation, migration, drug sensitivity, and immune evasion, have emerged as potential biomarkers and therapeutic targets, providing new directions for optimizing clinical management.

With regard to therapeutic targeting, m6A modifiers play crucial roles in targeted therapies. METTL3, as an oncogenic factor, enhances the proliferation and invasiveness of BC cells through m6A modifications. Inhibiting METTL3 can effectively suppress cancer cell proliferation and increase the efficacy of chemotherapeutic drugs. The METTL3-regulated miR-146a-5p and NUMB/NOTCH2 pathway offer a novel approach to targeted therapy. Interfering this pathway or reducing METTL3 expression could potentially inhibit the malignant behavior of BC cells.^13^ YTHDC1 has also gained attention for its roles in BC, where restoring YTHDC1 expression can increase BC cell sensitivity to chemotherapeutic agents such as cisplatin.^43^ Combining YTHDC1 modulation with autophagy inhibitors, such as chloroquine, may further suppress BC cell proliferation, providing a new strategy to enhance treatment efficacy.^17^

In addition, m6A-associated lncRNAs show significant potential in BC immunotherapy. The m6A-lncRNA risk scoring model, based on m6A-modified lncRNAs, effectively predicts patient responses to immune checkpoint inhibitors (e.g., programmed cell death protein 1 [PD-1]/programmed cell death ligand [PD-L] 1).^82^ Studies indicated that patients with low-risk scores generally exhibit higher responsiveness to immunotherapy, whereas those with high-risk scores often display immunosuppressive characteristics and weaker immune responses. This model offers a scientific basis for personalized immunotherapy, aiding in the identification of patients most likely to benefit from immune treatment and facilitating more precise therapeutic planning.

Finally, the potential application of m6A modifications in angiogenesis and metastasis control in BC is gaining interest. For instance, the m6A-modified lncRNA BLACAT3 promotes BC angiogenesis and hematogenous metastasis through NF-κB pathway activation. Research suggested that suppressing BLACAT3 expression or blocking its m6A modification stability can effectively reduce tumor angiogenesis and cancer cell invasiveness.^79^ These findings present new targets for anti-angiogenic therapy and offer innovative strategies for controlling BC metastasis (Table 3).

**Table 3:** Selected examples of ribonucleic acid modifications and their biological functions in BC

Modification	Proliferation	Migration/invasion	Distant metastasis	Drug sensitivity/resistance	Tumor microenvironment
m6A	METTL3 promotes proliferation through MYC, AFF4, and RELA signaling^18^	METTL3/YTHDF2 axis degrades tumor suppressors (SETD7, KLF4)^36^	GRIN2D stabilization by YTHDF1 promotes invasiveness^41^	YTHDF1 increases cisplatin resistance through PI3K/AKT pathway^24^	WTAP-mediated SYTL1 downregulation aids NK cell evasion^21^
m5C	NSUN2 stabilizes HDGF mRNA to promote proliferation^7^	ALYREF enhances migration through glycolysis (PKM2)^56^	High m5C levels correlate with increased invasiveness in MIBC^57^	YBX1 raises cisplatin resistance in BC^81^	ALYREF correlates with macrophage infiltration and immune modulation^56^
m1A	TRMT6/TRMT61A promotes stress resistance^78^	TRMT6/TRMT61A depletion reduces cell migration rate^78^	N/A	TRMT6/TRMT61A loss increases sensitivity to stress-inducing agents^78^	N/A
ac4C	NAT10 stabilizes p21 mRNA to promote proliferation^69^	Stabilization of SOX4 and AKT1 mRNAs promotes migration^71^	Stabilizes BCL9L to promote metastasis^71^	NAT10 enhances cisplatin resistance through AHNAK stabilization^70^	N/A
m7G	METTL1 promotes tumorigenesis through EGFR regulation^73^	METTL1 promotes invasiveness through miR-760 processing^75^	METTL1-mediated regulation of EFEMP1 promotes metastasis^73^	N/A	METTL1-regulated genes are linked to immune infiltration^74^

Abbreviations: ac4C: N4-acetylcytidine; AFF4: ALF transcription elongation factor 4; AHNAK: Neuroblast differentiation-associated protein; AKT: Protein kinase B; AKT1: Serine/threonine-protein kinase; ALYREF: Aly/REF export factor; BC: Bladder cancer; BCL9L: B-cell CLL/lymphoma 9 like; EFEMP1: EGF-containing fibulin-like extracellular matrix protein 1; EGFR: Epidermal growth factor receptor; GRIN2D: Glutamate [NMDA] receptor subunit epsilon-4; HDGF: Hepatoma-derived growth factor; KLF4: Krüppel-like factor 4; METTL: Methyltransferase-like protein; MIBC: Muscle-invasive bladder cancer; miR: MicroRNA; mRNA: Messenger RNA; m1A: 1-methyladenosine; m5C: 5-methylcytosine; m6A: N6-methyladenosine; m7G: 7-methylguanosine; NAT10: N-acetyltransferase 10; NK: Natural killer; NSUN2: NOP2/Sun RNA methyltransferase 2; N/A: Not available; PI3K: Phosphatidylinositol 3-kinase; PKM2: Pyruvate kinase M2; p21: Cyclin-dependent kinase inhibitor; RELA: v-rel avian reticuloendotheliosis viral oncogene homolog A; SETD7: SET domain containing lysine methyltransferase 7; SOX4: SRY-related HMG-box 4; SYTL1: Synaptotagmin-like protein 1; TRMT6: tRNA methyltransferase 6 non-catalytic subunit; TRMT61A: tRNA Methyltransferase 61A; WTAP: Wilms’ tumor 1-associating protein; YBX1: Y-box binding protein 1; YTHDF: YTH N6-methyladenosine RNA binding protein.

In conclusion, m6A modifications hold substantial promise for clinical applications in BC, particularly as diagnostic biomarkers, and in prognostic evaluation and targeted therapies. Continued exploration of m6A modification mechanisms and regulatory pathways will likely yield more effective strategies for the precision treatment of BC.

### 5.2. RNA modifications and the TME in BC

The TIM comprises cancer cells, immune cells, and the extracellular matrix, all of which play significant roles in tumor initiation and metastasis. Pan *et al*.^56^ demonstrated a significant correlation between ALYREF expression and infiltration of immune cells, particularly macrophages, in the TME. This finding suggests that ALYREF may influence BC progression and prognosis by modulating the TIM.

Li *et al*.^77^ used the ESTIMATE and CIBERSORT tools and found that tumors in the high-risk group had a greater composition of immune and stromal cells in the TME, indicating that m5C-related lncRNAs may affect BC prognosis by regulating the TME. In groups predicted to have a favorable prognosis, the expression levels of immune cells, including plasma cells, regulatory T cells, and CD8-positive T cells, were higher. This finding suggests that m5C-related lncRNAs may modulate tumor immune responses by influencing the abundance of specific immune cells.

Zhang *et al*.^72^ identified genes associated with m7G modification that were enriched in pathways such as Wnt signaling, ECM-receptor interaction, and the PI3K-Akt pathway, all being related to inflammation and tumor progression. Using the TIMER database, they assessed the association between four m7G modification-related prognostic genes and the infiltration of six immune cell types: B cells, CD4-positive T cells, CD8-positive T cells, neutrophils, macrophages, and dendritic cells. Furthermore, Ren *et al*.^74^ analyzed immune cell infiltration and immune checkpoint gene expression levels in high-risk and low-risk BC patient groups. They found that high-risk patients had elevated expression levels of PD-1, PD-L1, PD-L2, and CTLA-4, suggesting that m7G modifications may regulate the TIM and influence patient responses to immunotherapy.

### 5.3. Current status of research on RNA modification enzyme inhibitors and challenges

RNA modification enzymes, particularly RNA methyltransferases such as METTL3 and demethylases such as FTO, have emerged as promising therapeutic targets for cancer treatment. Pre-clinical studies have shown that FTO inhibitors, such as Rhein and meclofenamic acid (MA), have the potential to inhibit tumor growth. However, their specificity and potential off-target effects still present significant challenges.^83^ While Rhein demonstrated relatively low cytotoxicity against FTO, it also inhibits DNA repair enzymes such as ALKBH2 and ALKBH3, introducing potential off-target effects.^84^ In terms of treatment specificity, inhibitors such as MA have shown better selectivity for FTO inhibition, with minimal impact on other RNA-modifying enzymes. New-generation FTO inhibitors, including FB23-2 and Dac51, exhibit high selectivity, and *in vitro* studies have demonstrated promising anticancer activity. These inhibitors promote differentiation and apoptosis of acute myeloid leukemia (AML) cells by mimicking FTO depletion and enhancing CD8-positive T-cell immune functions.^83^,^85^

Furthermore, combining RNA modification enzyme inhibitors with conventional treatments, particularly chemotherapy and immune checkpoint inhibitors (e.g., anti-PD-1), has become an important area of research. Studies indicated that inhibition of METTL3 and METTL14 may sensitize cancers, including AML, to chemotherapy.^83^ Inhibition of METTL3 has also been shown to improve the efficacy of immune therapies, particularly in colorectal cancer and melanoma.^86^ However, the clinical application of RNA modification enzyme inhibitors faces several challenges, including drug delivery, personalized treatment strategies, and optimization of drug selectivity.

STC-15, the first METTL3 inhibitor to undergo a clinical evaluation, has been shown to have the potential to inhibit tumor growth through immune mechanisms. The ongoing Phase I clinical trial of STC-15 is assessing its safety and pharmacodynamics in solid tumor patients. At the 2024 American Society of Clinical Oncology Annual Meeting, interim clinical data on STC-15 in advanced cancer patients were reported, showing good tolerability and clinical activity, and providing valuable information to support the clinical application of RNA modification enzyme inhibitors.^87^

Overall, RNA modification enzyme inhibitors have a good prospect of being used for cancer treatment, but their specificity, off-target effects, and integration with existing treatment modalities warrant further research and optimization.

## 6. Future perspectives and challenges

In BC research, the extensive involvement of RNA modifications opens new avenues for unraveling molecular mechanisms and developing precision therapies. As a central RNA modification in BC research, m6A – alongside other modifications – impacts cancer cell biology through distinct molecular mechanisms, supporting various regulatory processes in BC. However, this diversity also adds complexity to research.

A critical goal for future studies will be to fully understand the interplay, coordination, and subtype-specific roles of different RNA modifications in BC. For example, the interactions between various RNA modifications and their combined effects within the same signaling pathway remain largely unexplored. Questions linger regarding whether modifications such as m1A, which regulates oncogene expression, or m5C, which stabilizes RNA and promotes protein synthesis, might work in tandem with m6A to drive cancer progression. In addition, the potential roles of pseudouridine modifications in RNA translation and splicing and their impact on BC cell adaptability and drug resistance represent crucial fields for further investigation.

For a clinical perspective, developing specific inhibitors or activators of RNA-modifying enzymes, as well as precision therapies targeting RNA modification-related gene expression, present considerable challenges. The diversity and complexity of RNA modifications render it difficult to precisely modulate specific modifications without impacting other physiological processes. Moreover, the development of individualized therapeutic approaches – including predicting the impact of various RNA modification combinations on patient outcomes – remains a significant challenge for clinical translation.

In summary, while RNA modifications in BC offer promising opportunities, great research effort has to be made to deepen our understanding and push forward the translation of these findings into effective clinical applications.

## 7. Conclusion

This review highlights the significant role of RNA modifications in the biology of BC. These modifications regulate essential processes such as cell proliferation, migration, drug resistance, and immune evasion, affecting the progression of BC. Targeting RNA modification pathways holds great promise as a strategy to enhance treatment efficacy and overcome drug resistance in BC therapy.

## Data Availability

Not applicable.
